# Numerical Analysis of Cement Placement into Drilling Fluid in Oilwell Applications

**DOI:** 10.3390/ma18133098

**Published:** 2025-06-30

**Authors:** Chengcheng Tao, Qian Wang, Goodarz Ahmadi, Mehrdad Massoudi

**Affiliations:** 1 School of Construction Management Technology, Purdue University, West Lafayette, IN 47907, USA; tao133@purdue.edu (C.T.); qianwang789@outlook.com (Q.W.); 2 Department of Mechanical and Aerospace Engineering, Clarkson University, Potsdam, NY 13699, USA; gahmadi@clarkson.edu; 3 Department of Mathematical Sciences, Carnegie Mellon University, Pittsburgh, PA 15213, USA

**Keywords:** well cementing, drilling fluid, VOF model, Herschel–Bulkley fluid

## Abstract

Understanding the displacement mechanism of cement slurry in drilling fluid is crucial for enhancing the safety of oil well cementing and mitigating geotechnical risks. This study investigated the oil well cementing process by simulating the displacement of drilling fluid by cement slurry in the annular space between the well casing and the surrounding formations using computational fluid dynamics (CFD). The volume-of-fluid (VOF) method in ANSYS-Fluent was employed to track the interfaces between drilling fluid, spacer fluid, and cement slurry. The study simulated fluid motion during drilling operations in the oil and gas industry, considering both smooth and irregular annular geometries around wells. The results show that the efficiency of cement slurry in displacing drilling fluid is higher in Case-2 (irregular outer walls) than in Case-1 (smooth outer walls). Under various inlet velocity conditions in Case-2, an optimal filling rate was achieved at an inlet velocity of 0.5 m/s. When the inlet velocity of the cement slurry was 0.2 m/s, a higher cement content was observed compared to 0.05 m/s, although some recirculation regions were more likely to form at this velocity.

## 1. Introduction

**Figure 1:**
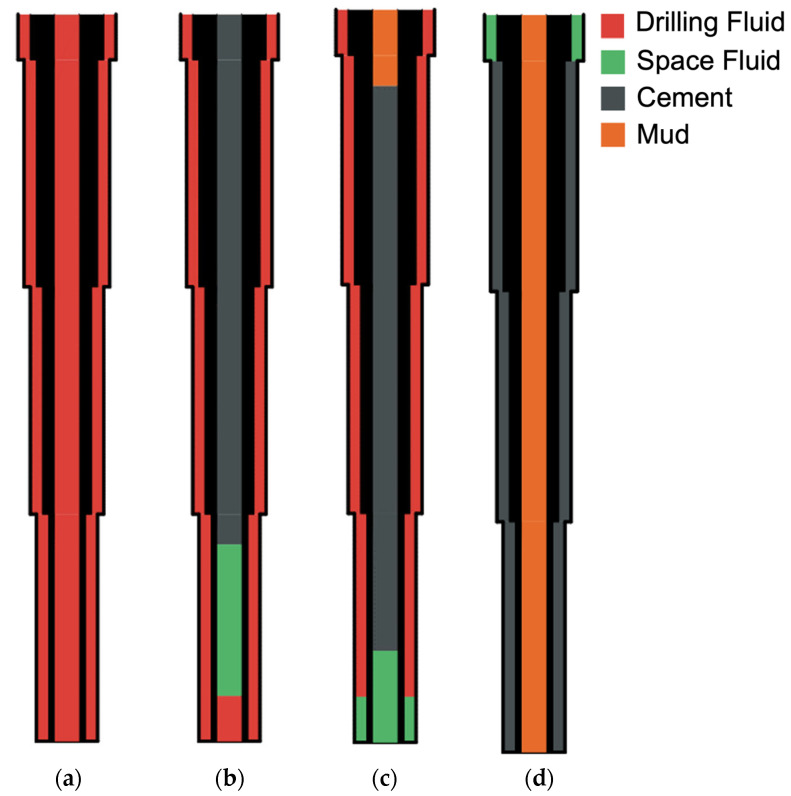
Schematic of primary cementing of a new casing at different stages. (**a**) initial stage; (**b**) intermediate stage; (**c**) intermediate stage; (**d**) completion stage.

Oil well cementing is one of the critical steps in the completion of oil and gas wells in the petroleum industry. This process is accomplished by pumping cement slurries into the annulus space between the casing and the geological formation surrounding the wellbore. Oil and gas wells are drilled at different stages. At the end of each stage, cement slurry is pumped into the annulus between the wellbore walls and the new casing located inside the wellbore extending from the surface to the end of the wellbore. A schematic of the primary cementing procedure at different stages is depicted in Figure 1. Usually, cement slurries are incompatible with drilling fluids, so a spacer fluid or a chemical wash compatible with the drilling fluid and the cement slurry is pumped first to form a layer between the drilling fluid and the cement slurry [1]. The spacer fluid prevents contamination of cement slurry with mud. It also improves the cleaning and removal of debris, mud cake, and drilling fluid from the inside of the casing. The annulus and its walls provide water-wet surfaces on the casing and the formation walls to create an effective bond at the casing–cement and cement–formation interfaces [2,3,4].

After the required amount of spacer fluid is placed, a cement slurry is pumped through the wellbore casing into the annulus space between the casing and the geological formation surrounding the wellbore [5,6]. When a sufficient amount of cement fills the annular space between the casing and the formation, the drilling fluid and other fluids are displaced in the annulus space. After completing each stage and setting the cement, the procedure is repeated to reach the desired location.

Primary cementing is one of the essential steps in the completion of oil and gas wells in the petroleum industry. The cementing is to support the wellbore and to provide a hydraulic seal on the outside of the steel tubes (casings and liners). Oil well cementing leads to the isolation of different fluid-bearing zones of the ground formation from one another and the surface [2]. However, since the early 1960s, the petroleum and cement industries have been challenged by fluid formation invasion into the pore spaces of cement lattices that can transfer to the surface, with subsequent negative economic and environmental impacts [7]. In recent years, several numerical and experimental investigations have been conducted to explore the causes of leakage and to provide possible solutions.

**Figure 2:**
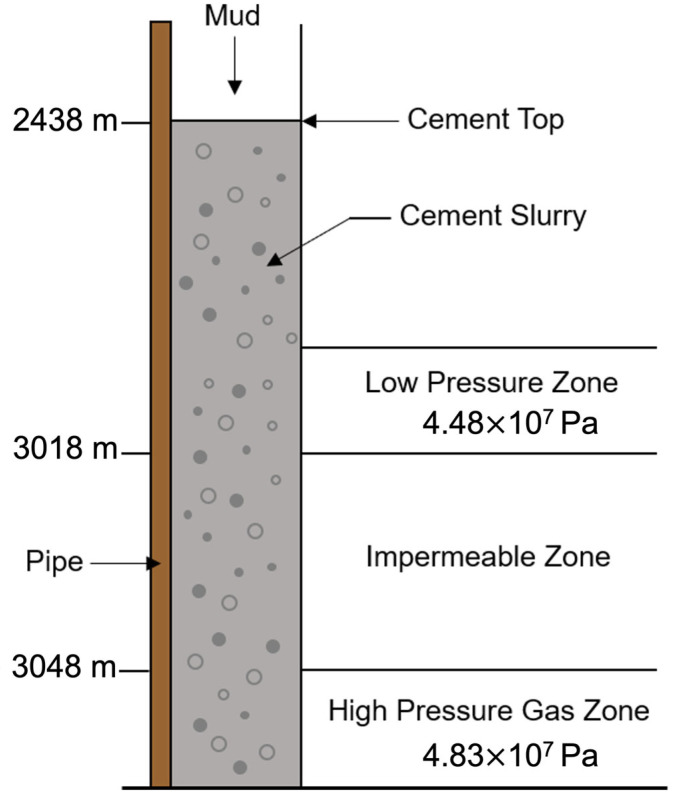
Schematic of the environmental conditions during oil well cementing.

Researchers have found that effective cleaning of the bore well is critical for a successful cementing job, since the integrity of the cement structure can be weakened by the porous media remaining after removing water from mud, debris, mud cake, and drilling fluid left in the annulus during cement setting [2,4,8]. Figure 2 shows a typical schematic of the environmental conditions during oil well cementing.

Since the cementing job is a complex process affected by various factors, performing numerical simulations in addition to laboratory and analytical investigations can be helpful in providing practical information for improved design and operation of the cementing process [9,10]. However, it is challenging to accurately evaluate the realistic influences of the critical parameters, such as the evolution of cement properties, the reduction in pore pressure, the quality of bonding at the interfaces (cement–casing and cement–formation), and the quality of fluid displacement, in numerical simulation of the cementing process. Therefore, this investigation was devoted to the development of a sophisticated numerical procedure that includes the primary parameters for accurate numerical simulations of the cementing process. To this end, the ANSYS-Fluent 2020 R2 was used to study the drilling fluid displacement and the cement slurry invasion of the annulus during the cementing process. At the same time, the empirical models of Beirute and Cheung [7] were included to incorporate the evolution of the hydrostatic and pore pressures during the process. In addition, to explore the effects of wall roughness and the fluid spacer, numerical simulations were conducted for different annulus geometries with irregular/flat formation surface shapes and various conditions for the flow of cement slurry. Tao et al. [5,6,11,12,13] reviewed the rheological models of oil well cement slurries and proposed a new model, which is based on a modified form of the second-grade non-Newtonian fluid.

Previous researchers have made many contributions to the understanding of the cementing process. However, there are still research gaps in simulating the cement–drilling fluid replacement process. This study provides a computational analysis of the cement–drilling fluid replacement process in the oil and gas drilling industry. The cement slurry is modeled as a Herschel–Bulkley fluid, and the multiphase flow during the replacement process is simulated using the VOF method of the ANSYS Fluent code. In the subsequent sections, we present the fundamental governing equations and constitutive relations used for the cement slurry in our simulation, describe the detail of the system geometry designed in SolidWorks 2021, and present the computational mesh. We also show simulation results of cement slurry injection into drilling fluid (water) in the 3D annulus around the well with flat and irregular walls and investigate the replacement process under different inlet velocities. The effects of the geometry and inlet speeds on cement replacement rates are examined for different annulus models. The presented numerical simulations reveal the nature of the replacement rate and flow patterns in the cement–drilling fluid replacement process. The findings provide technical support and practical engineering guidance for the oil well cementing process.

## 2. Governing Equations and Constitutive Relations

**Figure 3:**
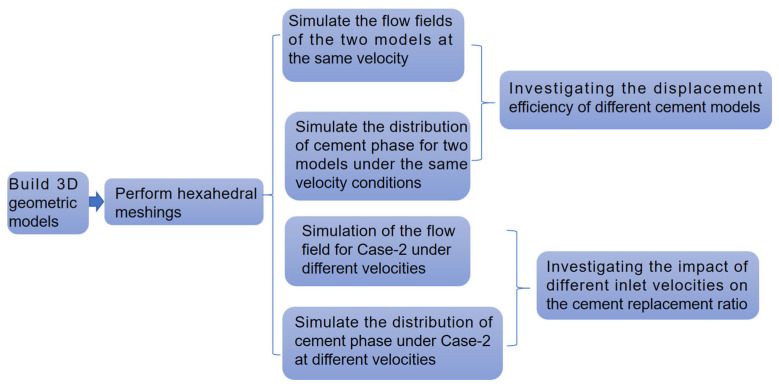
Schematic of the simulation process.

Cement is a complex multiphase material whose microstructure and interwoven chemical and physical properties pose significant challenges in establishing its constitutive model. A cement slurry, primarily composed of cement particles, water, and air, exhibits pronounced non-Newtonian characteristics, including shear-thinning effects, thixotropy, and yield stress [14]. In this study, we investigated the flow behavior of a cement slurry at different velocities to achieve effective cementing. Cement exhibits complex rheological properties, behaving as a visco-plastic or visco-elastic material in its paste form and transitioning to a nonlinear material after hardening. The most commonly used rheological models for cement paste are the Bingham model [15] and the Herschel–Bulkley model [16]. To mathematically model the flow of cement slurry at various velocities, a two-phase (or multiphase) approach can be considered. In this approach, separate constitutive relations are required for the stress tensors, $T_{1}$ and $T_{2}$, of the cement slurry and drilling fluid, respectively. Additionally, volume fractions, $\phi_{1}$ and $\phi_{2}$, for each phase and the interaction forces, $f_{I}$, between them need to be taken into account. The volume-of-fluid (VOF) approach was particularly suitable for our study, as it is computationally feasible and provides a robust framework for studying the multiphase flow of cement slurry at different velocities. Therefore, in this paper, we utilized the VOF approach to computationally investigate the flow of a cement slurry for the oil well cementing process and its optimization. Figure 3 shows a schematic of the entire simulation process in this thesis.

### 2.1. Governing Equations

We applied governing equations, including continuity and momentum equations, to simulate the movement of cement displacing drilling fluid. The volume-of-fluid (VOF) approach was used to investigate the interfaces between different phases (drilling fluid, spacer fluid, cement slurry, and the formation fluid).

At any point x∈Ω and time $t\left(>0\right)$, the cement slurry and the drilling fluid are considered as a “fluid mixture” with the velocity $u\left(x,t\right)$ and density $\rho \left(x,t\right)$. This “fluid mixture” is considered to be incompressible:

(1) ∇·u=0

where div stands for the divergence operator. The density of the “fluid mixture” is defined using the volume fraction of the cement slurry, ϕ(x,y).

(2) $$\rho \left(x,t\right)=\phi \rho_{1}+\left(1-\phi \right)\rho_{2}$$

where $\rho_{1}$ and $\rho_{2}$ are the constant densities of the cement slurry and the drilling fluid, respectively.

The balance of momentum equation is given as

(3) $$\frac{\partial }{\partial t}\left(\rho u\right)+\nabla \cdot \left(\rho uu\right)=\nabla \cdot T+\rho b+⟦\Sigma ⟧$$

where b is the body force, T is the stress tensor, and $⟦\Sigma ⟧$ is the term related to the interfacial interaction between the cement slurry and the drilling fluid.

The stress tensor in the VOF approach of “fluid mixture” is described through the pressure (p) and the viscosity (μ), which is dependent on the volume fraction, shown in Equation (4).

(4) $$\mu \left(x,t\right)=\phi \mu_{1}+\left(1-\phi \right)\mu_{2}$$

where $\mu_{1}$ and $\mu_{2}$ are the viscosities of the cement slurry and the drilling fluid, respectively. At any point x∈Ω in time t>0, the Cauchy stress tensor for the “fluid mixture” is as follows:

(5) $$T\left(x,t\right)=-p\left(x,t\right)I+\mu \left(x,t\right)A_{1}$$

where I is the identity tensor, $A_{1}$ is $A_{1}=\nabla u+{\left(\nabla u\right)}^{T}$ =2D, and D is the symmetric part of the velocity gradient. Here, ∇ is the gradient operator.

The volume fraction of cement slurry, ϕ, which serves as a flag identifying the presence of a given fluid phase in the mixture, is governed by a convection–diffusion-type equation. That is,

(6) $$\frac{\partial \phi }{\partial t}+u\cdot \nabla \phi =0\;$$

### 2.2. Constitutive Relations

#### 2.2.1. Interfacial Interaction Between the Two Fluids

The variations in physical properties (e.g., density and viscosity) between the two immiscible fluids lead to interfacial interactions. The net tensile force acting on the interface, characterized by curvature κ and separating the two fluid phases, can be modeled as follows [17]:

(7) $$⟦\Sigma ⟧=\int_{V\left(t\right)}\sigma \kappa \left(x\right)n\left(x\right)\delta \left[n\left(x_{s}\right)\cdot \left(x-x_{s}\right)\right]dx$$

(8) $$⟦\Sigma ⟧≅\sigma \kappa \left(x_{s}\right)n\left(x_{s}\right)$$

where σ is the surface tension, n is the unit normal vector to the interface, and δ is the Dirac function.

The curvature, κ, of a surface at a point $x_{s}$ along the unit vector $\hat{n}$ to the interface is assumed to be related to $\hat{n}$:

(9) $$\kappa =-\nabla \hat{n}$$

(10) $$\hat{n}=\frac{\nabla \phi }{\left|\nabla \phi \right|}$$

According to Brackbill et al. [17], due to computational challenges, the normal vector **n** in Equation (8) can be effectively substituted by −∇ϕ. This allows the source term associated with the interfacial interaction term to be expressed by

(11) $$⟦\Sigma ⟧=\sigma \left[div\left(\frac{\nabla \phi }{\left|\nabla \phi \right|}\right)\right]\nabla \phi$$

We should mention that this interaction term is just one of the various interaction forces, taking the form of Equation (12), along with other interaction forces such as drag, lift, etc. [18].

(12) $$⟦\Sigma ⟧=\omega \nabla \phi$$

#### 2.2.2. Stress Tensor

We consider a scenario where a cement slurry (fluid 2) is injected into drilling fluid (fluid 1). The densities of the cement slurry ($\rho_{1}$) and drilling fluid ($\rho_{2}$) are assumed to be constant, as specified in Equation (2). In general, as shown by Tao et al. [6,11], a cement slurry may exhibit various nonlinear effects such as yield stress, thixotropy, shear-dependent viscosity, concentration-dependent viscosity, etc. In this paper, we assume that the slurry can be modeled as a Herschel–Bulkley fluid (the effects of thixotropy and concentration-dependent viscosity are ignored). Thus, the stress tensor in the “fluid mixture” of the VOF method is given by

(13) $$T\left(x,t\right)=-p\left(x,t\right)I+\tau \left(x,t\right)=-p\left(x,t\right)I+\tau_{1}\left(x,t\right)+\tau_{2}\left(x,t\right)$$

where $\tau_{1}$ and $\tau_{2}$ are the stress tensors for the cement slurry and the drilling fluid, respectively.

We assume that the cement slurry behaves as a non-Newtonian fluid [5,6,11,12,13], and we use the Herschel–Bulkley [19] model for the cement slurry [20,21,22]. According to this model, the shear stress τ is given as

(14) $$\tau =\tau_{y}+k{\dot{\gamma }}^{n}$$

where $\dot{\gamma \;}$ is the shear rate. According to Banfill [23], k is 2.5 or 0.25 and n is 0.75 or 1.25 for cement. For n = 1 and $\tau_{y}=0$, the fluid becomes a Newtonian fluid.

The viscosity associated with the Herschel–Bulkley model is

(15) $$\mu =\frac{\tau_{y}}{\dot{\gamma \;}}+k{\left(\frac{\dot{\gamma }}{\dot{\gamma_{c}}}\right)}^{n-1}\;\mathrm{if}\;\dot{\gamma }<\dot{\gamma_{c}}$$

(16) $$\mu =\frac{\tau_{y}\left(2-\frac{\dot{\gamma \;}}{\dot{\gamma_{c}}}\right)}{\dot{\gamma_{c}}}+k\left[\left(2-n\right)+(n-1)\frac{\dot{\gamma \;}}{\dot{\gamma_{c}}}\right]\;\mathrm{if}\;\dot{\gamma }>\dot{\gamma_{c}}$$

where $\dot{\gamma_{c}}$ is the critical shear rate.

Table 1 shows the properties of the cement slurry and the drilling fluid used in this study.

### 2.3. Assumptions and Boundary Conditions

We made the following assumptions: (1) the cement and the drilling fluids are incompressible and homogenous viscous fluids; (2) we also neglected the heat transfer effects, i.e., we assumed an isothermal condition. To simplify the model and focus on the fluid dynamic behavior, we assumed a constant temperature throughout the cementing process. In real-world oil well cementing, the process is usually performed in a short time with limited temperature changes. This results in negligibly small effects of temperature variation on the viscosity and flow characteristics of cement slurry. Using the isothermal flow assumption reduces model complexity and focuses our study on the fluid dynamics of the process.

The initial conditions are as follows:

(1) The mass fraction of the drilling fluid in the entire calculation domain is 1.
(2) The mass fraction of the cement slurry is 0.

The boundary conditions are as follows:

(1) Inlet boundary condition: For the cement slurry inlet velocity, which is downward, we used the following values: 0.5 m/s, 0.2 m/s, and 0.05 m/s.
(2) Wall boundary condition: The walls are standard walls with no slip.
(3) Outlet condition: Outflow.

## 3. Geometry and Mesh

**Figure 4:**
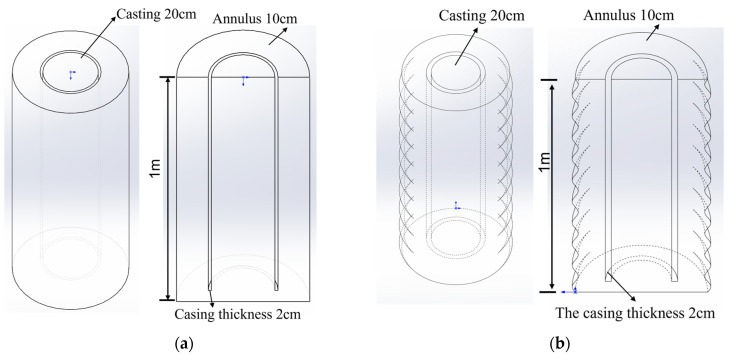
Three-dimensional oil well cementing cases: (**a**) Case-1 with a smooth wall surface; (**b**) Case-2 with a rough wall surface.

To simulate the actual drilling conditions, we used SolidWorks software to create two different oil well annulus conditions for the smooth and rough outer walls to simulate the fluid motion during the cementing process. As shown in Figure 4a, a three-dimensional (3D) cylindrical system was created with a smooth wall surface. Figure 4b shows a 3D case with a rough wall surface. The case for the rough wall surface was expanded from the case with the smooth wall surface by adding a layer of irregular (wavey) walls. Thus, the volume of the annulus with the rough wall surface is larger than that of the smooth wall surface. Figure 4 shows the system in full and cross-sectional views. The length of the pipe segment is 1 m, the casing diameter is 20 cm, the casing thickness is 2 cm, and the annulus thickness is 10 cm.

**Figure 5:**
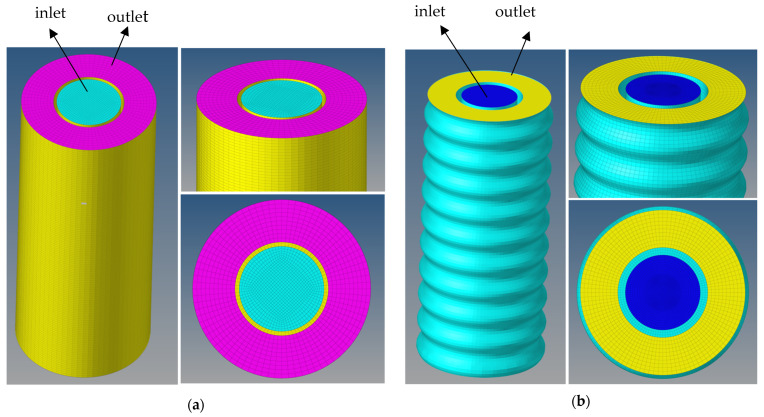
Meshing of the 3D oilwell cementing: (**a**) Case-1; (**b**) Case-2.

To ensure the accuracy of the numerical simulation, we employed a hexahedral meshing strategy with a mesh quality above 0.7 and a surface mesh length of 3 mm. We utilized the finite volume method in Ansys Fluent to discretize and solve the governing equations, achieving efficient computation while meeting the accuracy requirements. After extensive testing, a time step of 0.0001s was selected to ensure stable simulation results and high computational efficiency. The two meshes were imported into HyperMesh for generation. Figure 5 displays the hexahedral meshes for Case-1 and Case-2. As shown, cement is injected through the inlet to displace the drilling fluid, with the outlet positioned on the same horizontal plane as the inlet.

## 4. Numerical Results and Discussion

### 4.1. Mass Fraction of Cement Phase in Different Cases Under Uniform Velocity Conditions

**Figure 6:**
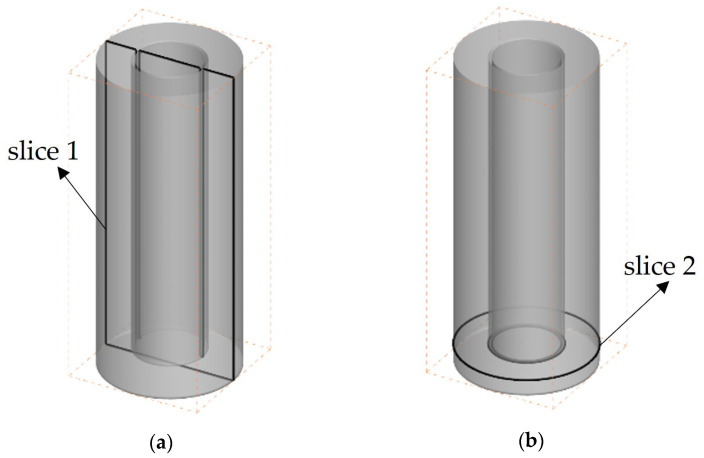
Two sections for monitoring the simulations of Case-1: (**a**) slice 1—longitudinal section; (**b**) slice 2—lateral section.

We investigated the 3D flow in the irregular and the flat wall configurations to simulate the cement slurry injection into the drilling fluid during the cementing process. Figure 6 displays the slices at two primary locations of Case-1 discussed in this paper, specifically the central longitudinal section, slice 1, and the lateral slice 2. Similarly, Case-2 also has sections cut at the same positions.

**Figure 7:**
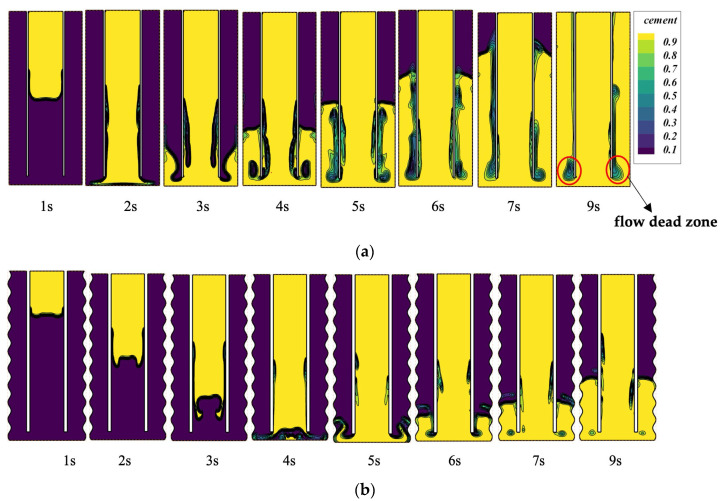
The mass fraction of cement at slice 1 at different times: (**a**) Case-1 with smooth outer wall; (**b**) Case-2 with irregular wall.

We used the Herschel–Bulkley fluid model for cement slurry in a 3D cylinder with flat walls (Case-1) and irregular walls (Case-2). Figure 7a,b illustrate the filling of cement at different times for both situations after the casing has been set in the well and the cement slurry has been injected through the casing at a velocity of 0.5 m/s.

Figure 7 shows the mass fraction of cement at slice 1 at different times. Compared to Figure 7a, the larger annulus volume in Figure 7b has altered the flow resistance. Additionally, the larger volume makes it more likely for the fluid to generate vortices at slice 2. Figure 6a illustrates the flow conditions for the cement slurry at slice 1 in Case-1. After the cement enters, it flows vertically downward along the casing. At slice 2, a larger swirling flow is observed in this area. Due to the swirl formation, a “dead flow zone” is prone to form in this area, which is an area where the rate of displacement of drilling fluid by cement slurry is very low. Figure 7b shows the flow of cement slurry in the annulus with irregular wall surfaces. Compared to Figure 7a, we can see that the flow state of the cement slurry near slice 2 has changed, with a smaller swirl formed and a higher mass fraction of the cement phase than in Figure 7a.

The comparison of the two cases indicates that the geometric configuration of the wall substantially influences the flow behavior of cement slurry. Smooth-walled scenarios are more likely to experience flow asymmetry and form dead zones. By contrast, irregular-walled scenarios may improve flow and increase cement slurry displacement efficiency. These differences arise from variations of initiation in two-phase flow instability under different wall conditions. To fully understand this phenomenon, additional studies will be conducted in future works.

**Figure 8:**
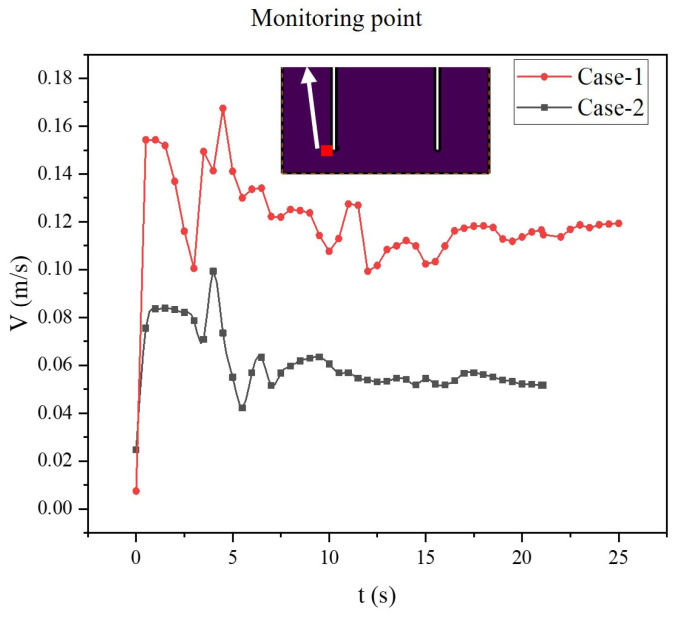
The velocity variation curves at the monitoring point for the two cases.

During the process of cementing for the case in which the slurry enters at 0.5 m/s from the inlet and fills the entire gap, the flow velocity at a point at the bottom of the annulus outlet is monitored. The monitoring point is located 1 mm away from the casting wall. Due to the casting wall hindering cement flow, the velocity at this point is significantly lower than the inlet velocity of 0.5 m/s. Figure 8 compares the time variation of the velocities for the two cases. It can be seen that the flow velocity increases sharply at the beginning and then fluctuates at different plateau levels. Under the same inlet velocity conditions, the annulus volume (mean cross-section area) in Case-2 with the rough wall is greater than that of Case-1 with a smooth wall. At the same inlet condition, there is a higher likelihood of forming recirculation regions at Case-1, resulting in a lower velocity at the monitoring point. As a result, the velocity at the monitoring point in Case-2 is lower than in Case-1, indicating that Case-2 is less likely to form a dead flow zone in this area. The two curves in Figure 8 have similar fluctuation trends, from which we could conclude that both cases involve the formation and destruction of recirculation regions in the annulus region.

**Figure 9:**
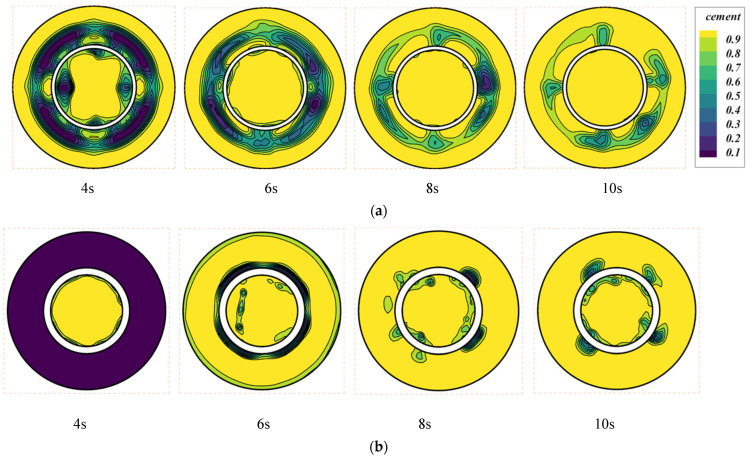
The mass fraction contours of cement at slice 2 at different times: (**a**) Case-1 with smooth outer wall; (**b**) Case-2 with irregular wall.

Figure 9 presents the volume fraction contours of the cement phase at slice 2 for both cases at different times. From 4 s to 10 s, the overall rate of displacement of drilling fluid by the cement slurry in Case-1 is smaller than that in Case-2 at this slice position. Comparing the situation at 6 s, the mass fraction of cement slurry on the outer wall of Case-2 is greater than in Case-1, indicating that the uniformity of the cement slurry in Case-2 is better at this time.

**Figure 10:**
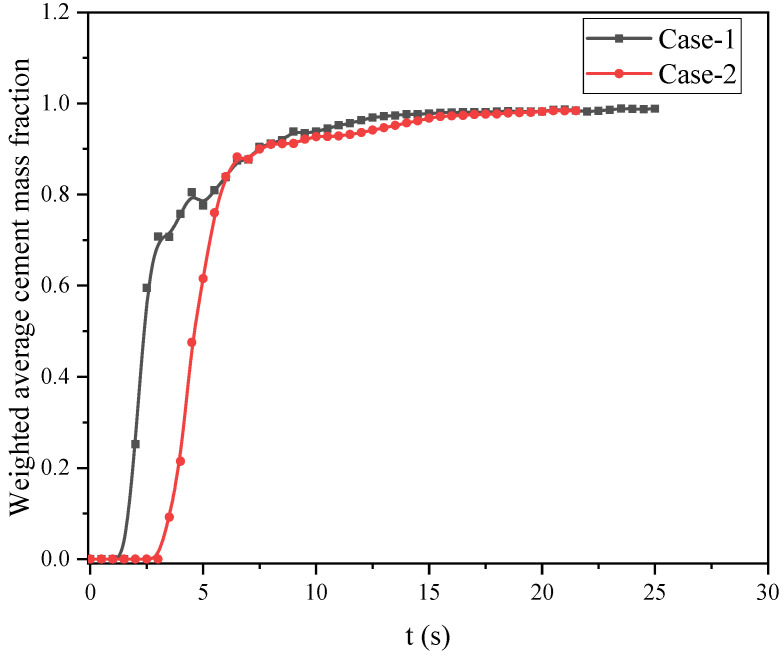
Curves of the weighted average mass fractions of the cement at slice 2 for the two cases.

To further understand the impact of cement slurry replacing drilling fluid at slice 2, we also performed a surface-weighted average of the mass fraction of the cement phase on this slice, and the results are shown in Figure 10. We can see that from 0s to 2.5 s, the weighted average mass fractions of the cement phase on the slice for both Case-1 and Case-2 behave differently. For Case-2, the surface-weighted slurry mass average remains at zero for up to 2.5 s. It then rises sharply between 2.5 and 5 s. At 5 s, the values for both cases become the same and remain consistent afterward. However, during the period from 2.5s to 6s, the weighted mass fraction on the surface fluctuates smoothly. Notably, Case-1 exhibits a small amplitude fluctuation zone, indicating that the cement slurry and drilling fluid alternated during this time, while Case-2’s curve had no significant fluctuation during this stage, indicating that the replacement rate of the cement slurry is more uniform. After 6s, the curves of the two cases become consistent and coincide, indicating that the cement slurry has completely replaced the drilling fluid.

In summary, having an irregular outer wall surface with a larger annular cross-section area improves the filling of cement slurry. Through the analysis of the weighted average mass on the surface of slice 2, it was found that when the outer wall surface changes from smooth and regular to irregular, the efficiency of cement replacement is improved. In the next section, we describe a study on different inlet velocities for Case-2 with an irregular outer wall surface.

### 4.2. Variation of Cement Phase in Case-2 Under Different Inlet Velocities

**Figure 11:**
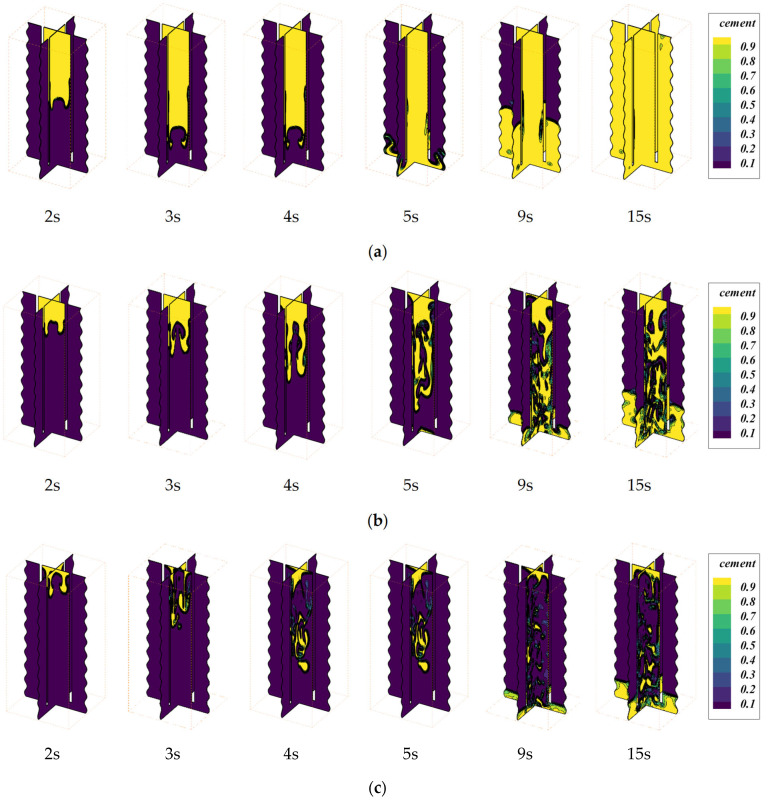
Volume fraction contours are shown on two vertical planes that are perpendicular to each other: (**a**) inlet velocity: 0.5 m/s; (**b**) inlet velocity: 0.2 m/s; (**c**) inlet velocity: 0.05 m/s.

Based on previous studies of cement slurry, we observed that the filling of cement slurry in the annular region is better in Case-2. Therefore, we performed additional simulations for Case-2, examining the flow behavior of cement slurry for inlet velocities of 0.5, 0.3, and 0.05 m/s over various time periods, and the results are illustrated in Figure 11.

It can be observed that at an inlet velocity of 0.5 m/s, a higher flow rate results in minimal intermingling of cement slurry and drilling fluid during the infusion process. Figure 11b corresponds to an inlet velocity of 0.2 m/s, where it is evident that between 2s and 5s post-infusion, the cement slurry, due to its reduced velocity, adheres to the periphery with diminished central flow. Beyond 5s in the casing region, a pattern of alternating cement slurry and drilling fluid layers is discernible, with vortices of cement slurry forming near slice 2, indicative of a two-phase flow. Compared to Figure 11a, the filling efficiency in Figure 11b is significantly inferior. Figure 11c shows the volume fraction contours for an inlet velocity of 0.05 m/s, where the reduced velocity of the cement at the inlet causes the cement to move downward along the wall slowly. At 9 s, a comparison between Figure 11b and Figure 11c reveals that the flow beneath slice 2 in Figure 11c exhibits a lower velocity. The reason is that the inlet velocity in Figure 11c is lower, resulting in slower flow and, consequently, a lower turbulent kinetic energy of the cement at the bottom. Therefore, the cement filling at the bottom in Figure 11c is better than that in Figure 11b, and the “dead flow zone” is minimal.

**Figure 12:**
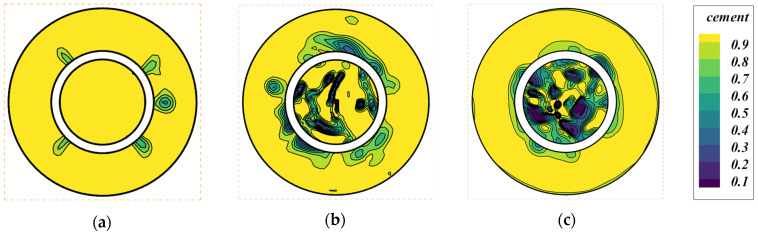
The cement phase contour at slice 2 of Case-2 at 20 s for different velocities: (**a**) inlet velocity: 0.5 m/s; (b) inlet velocity: 0.2 m/s; (**c**) inlet velocity: 0.05 m/s.

In our analysis of Case-2 at slice 2, Figure 12 illustrates the cement phase contours at 20 s. By comparing these three figures, we can observe that the cement slurry with an inlet velocity of 0.5 m/s replaces the drilling fluid at the fastest rate. Similarly, the cement slurry with an inlet velocity of 0.05 m/s replaces the drilling fluid at the slowest rate. Additionally, we can find that the uniformity of cement within the annular region in Figure 12a,c is higher than that in Figure 12b. The smaller the cement phase fraction within the annular region, the more likely it is that a velocity recirculation zone will form in this area, resulting in reduced cement displacement efficiency.

**Figure 13:**
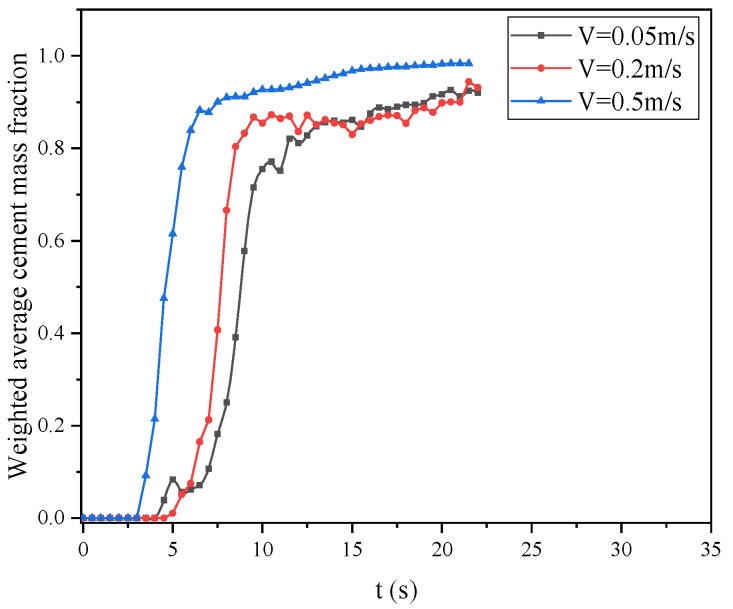
Curves of the weighted average mass fractions of cement at slice 2 versus time for different cement inlet velocities for Case-2.

At the position of slice 2 in Case-2, we made a surface-weighted average cement volume fraction in this section, as shown in Figure 13. According to these curves, between 6 s and 12.5 s, the cement content is significantly higher when the cement inlet velocity is 0.5 m/s compared to the other two velocities, with the lowest cement content observed at an inlet velocity of 0.05 m/s. However, after 12.5 s, we can observe slight fluctuations in the curves for cement slurry inlet velocities of 0.2 m/s and 0.05 m/s. It can be seen that at an inlet velocity of 0.2 m/s, the cement content on slice 2 is higher than that at an inlet velocity of 0.05 m/s. Therefore, we can conclude that the optimal inlet velocity for the cement slurry is 0.5 m/s. At an inlet velocity of 0.2 m/s, recirculation regions are more likely to form.

### 4.3. The Velocity Vector Fields for Different Cases

**Figure 14:**
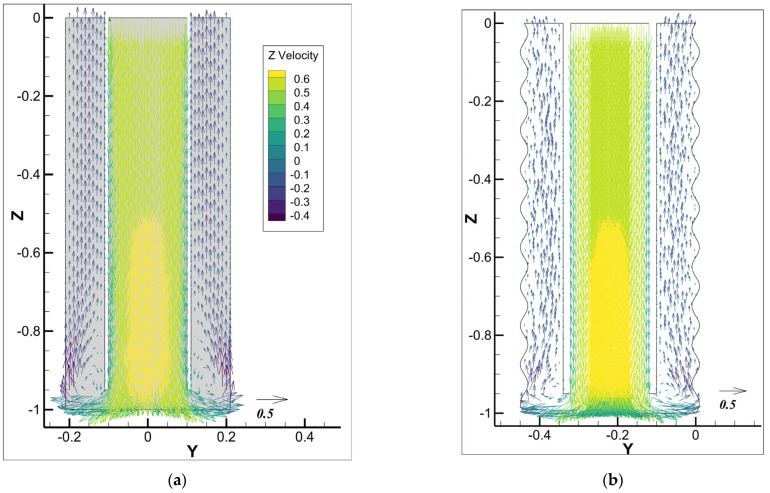
Velocity vector field colored with the mixture z-velocity for different cases for the cement slurry inlet velocity of 0.5m/s: (**a**) Case-1 with smooth outer wall; (**b**) Case-2 with irregular wall.

Figure 14 presents the velocity vector field colored by the z-velocity of the mixture at 20 s after injection for an inlet cement slurry velocity of 0.5m/s for different cases. In Case-1, with a smooth outer wall, a pronounced recirculation region near the wall forms at the bottom of the annulus, characterized by smaller velocity magnitudes near the eddy region. This causes flow stagnation and inefficient cement replacement. In Case-2, with an irregular outer wall and larger average cross-section, the average upward velocity is lower than in Case-1 for the smooth wall. However, the bottom recirculation region is significantly reduced, resulting in a more uniform velocity distribution and relatively larger velocity magnitudes near the wall. This observation suggests that the irregular outer wall diminishes recirculation region formation and boosts drilling fluid displacement efficiency.

**Figure 15:**
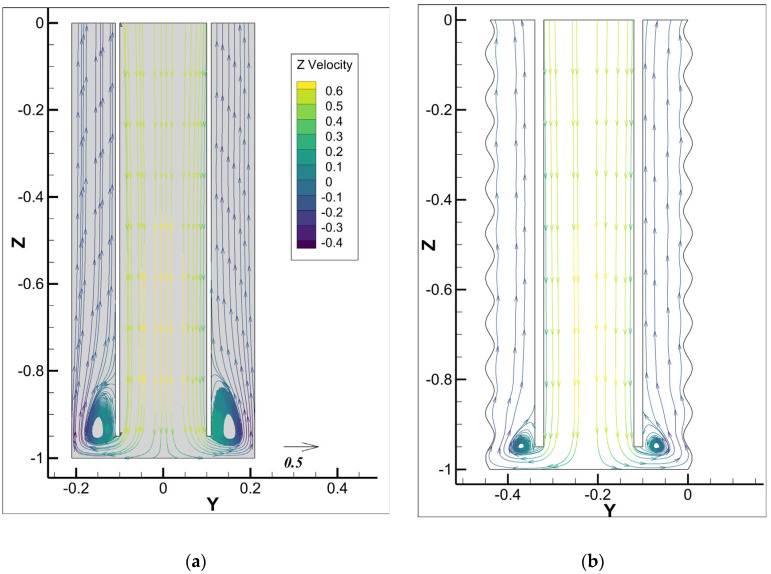
Mixture flow streamlines for different cases for the cement slurry inlet velocity of 0.5m/s: (**a**) Case-1 with smooth outer wall; (**b**) Case-2 with irregular wall.

Figure 15 presents the mixture flow streamlines at slice 1, colored by the z-velocity of the mixture at 20 s after injection. Case-1, with the smooth outer wall, leads to higher velocity in the annulus compared to Case-2 with the rough wall. In Case-1, however, a large recirculation region is formed at the bottom as the flow enters into the annulus region, with reversed flow generating a flow stagnation region and inefficient displacement. In Case-2, the irregular outer wall with a larger average cross-section reduces the recirculation region’s size and raises its position, resulting in more uniform streamlines and smoother velocity changes. This observation shows that an irregular outer wall with a larger average cross-section can improve the flow into the annulus by reducing the recirculation regions.

Table 2 compares the performance of Case-1 (smooth outer wall) and Case-2 (irregular outer wall) across different parameters based on the findings in Figure 7, Figure 9, Figure 14, and Figure 15. From the mixture velocity information, the annulus filled more quickly by cement slurries has a higher cement displacement efficiency. Case-1 has a lower displacement efficiency, a uniform and symmetric velocity distribution, and significant recirculation region formation as the flow enters the annulus. Case-2 features a higher replacement efficiency, a non-uniform velocity distribution with local variations, and a smaller recirculation formation at the entrance to the annulus. Both cases share an optimal inlet velocity of 0.5 m/s, which is the highest inlet velocity studied. This observation indicates that the outer wall’s smoothness and annulus cross-section significantly impact cement slurry replacement efficiency. An irregular outer wall with a larger cross-section boosts replacement efficiency but also complicates velocity distribution and recirculation region formation. In particular, a larger annulus cross-section at the bottom would be beneficial in the cementing process.

Case-2’s results indicate that irregular outer walls with larger annulus volumes enhance cement slurry replacement efficiency. In practice, complex geological formations generally have irregular outer walls. However, the increased recirculation region formation in Case-2 at the velocity of 0.2 m/s in the annular region suggests a need for caution to prevent potential flow instabilities (Figure 12). The flow patterns observed in Case-2 are scalable to larger annular spaces, provided the geometric complexity is similar. This scalability is supported by the consistent velocity profiles observed across different time points, implying that the fundamental flow dynamics remain unchanged with scale.

## 5. Conclusions

In this paper, we simulated fluid motion during drilling in the oil and gas industries. We created the geometry of the system in SolidWorks and used the ANSYS-Fluent code with the volume-of-fluid (VOF) method to solve the governing equations. We applied the Herschel–Bulkley model to simulate the movement of cement slurry into drilling fluid (water) in a 3D segment of a well with a flat and irregular annulus. This study shows that the efficiency of cement replacing drilling fluid in Case-2 with irregular outer walls and a higher annulus volume is greater than that in Case-1 with smooth outer walls. Under different inlet velocity conditions in Case-2, it was found that the filling rate is optimal when the inlet velocity is 0.5m/s. When the cement inlet velocity is 0.2 m/s, the cement content is higher than that at an inlet velocity of 0.05 m/s. However, recirculation regions are more likely to form on slice 2 at this velocity.

To simplify the model and focus on the fluid dynamic behavior, we assumed a constant temperature throughout the process. Temperature variations can affect the viscosity and flow characteristics of cement slurry. However, in actual oil well cementing operations, the entire process is typically completed in a relatively short time, and temperature changes are relatively small. Therefore, we assumed that the temperature remained constant throughout the process, that is, an isothermal flow. This assumption simplified the model and allowed us to concentrate more on the study of fluid dynamic behavior. Nevertheless, we recognize that in some cases temperature changes may have a noticeable impact on the flow behavior of the cement slurry. Therefore, we have discussed the limitations of this assumption in detail in the Discussion section and suggest further exploration of the impact of temperature changes on the cementing process in future studies.

Furthermore, for future studies, we plan for additional simulations to include the effect of variation in the hydrostatic and pore pressures and the migration of formation fluid to the annulus. In addition, we propose to conduct simulations using the Large Eddy Simulation (LES) method in the Fluent model. LES is capable of capturing large-scale turbulent eddies while modeling small-scale turbulence, thereby more accurately reflecting the complexity of the flow. Through this approach, we can better understand the interaction between drilling fluid and cement slurry under high-velocity conditions, as well as the nature of asymmetry formation of slurry flow during cementing. This understanding will provide a more scientific basis for optimizing the displacement efficiency of cement slurry.

## Figures and Tables

**Table 1:** Fluid properties of cement slurry and drilling fluid.

Liquid	Cement Slurry	Drilling Fluid
Density (kg/m^3^)	1200	998
Surface Tension (N/m)	0.07	0.07
Rheological Model	Herschel–Bulkley	Linear Newtonian
Viscosity (kg/ms)	N/A	${1\times 10}^{-3}$
Consistency Index, k (kg∙s ^n−2^/m)	0.6	N/A
Flow Index, n	0.4	N/A
Yield Shear Stress, $\tau_{y}$ (Pa)	1.4	N/A
Critical Shear Rate, $\dot{\gamma_{c}}$ (1/s)	5.5	N/A

**Table 2:** Structured comparison of Case-1 and Case-2.

Parameter	Case-1 (Smooth Outer Wall)	Case-2 (Irregular Outer Wall)
Replacement Efficiency	Lower	Higher
Velocity Distribution	Uniform and symmetric	Non-uniform with localized variations
Recirculation Region Formation	large	Small
Optimal Inlet Velocity	0.5 m/s	0.5 m/s

## Data Availability

The original contributions presented in this study are included in the article. Further inquiries can be directed to the corresponding author.

## Nomenclature

Symbol	Description
T	Cauchy stress tensor of the “fluid mixture”
$A_{1}$	Kinematical tensor
D	Symmetric part of the velocity gradient tensor
I	Identity tensor
$\tau_{1}$	Cement slurry phase stress tensor
$\tau_{2}$	Drilling fluid phase stress tensor
$⟦\Sigma ⟧$	Interfacial interaction between the two phases
${II}_{2D}$	Second invariant of the tensor 2D
${II}_{\tau_{1}}$	Second invariant of the stress tensor $\tau_{1}$
p	Pressure of the “fluid mixture”
u	Velocity of the “fluid mixture”
$u_{1}$	Velocity of the cement slurry phase
$u_{2}$	Velocity of the drilling fluid phase
$u_{r}$	Relative velocity between the two phases
n	Unit normal vector
k	Consistency of the cement slurry (for Herschel–Bulkley fluid)
n	Power-law exponent of the cement slurry (for Herschel–Bulkley fluid)
ϕ	Volume fraction of the cement slurry
σ	Surface tension
κ	Curvature of the interface between the two phases
δ	Dirac function
$\tau_{y}$	Yield stress of the cement slurry
μ	Viscosity of the “fluid mixture”
$\mu_{1}$	Viscosity of the cement slurry phase
$\mu_{2}$	Viscosity of the drilling fluid phase
ρ	Density of the “fluid mixture”
$\rho_{1}$	Density of the cement slurry phase
$\rho_{2}$	Density of the drilling fluid phase
∇	Gradient operator
